# Inferring and quantifying causality in neuronal networks

**DOI:** 10.1186/1471-2202-12-S1-P192

**Published:** 2011-07-18

**Authors:** Daniel Chicharro, Ralph G Andrzejak, Anders Ledberg

**Affiliations:** 1 Department of Information and Communication Technologies, Universitat Pompeu Fabra, Barcelona, Catalonia, 08018, Spain

## 

The idea of inferring causal interactions from a variable X to another variable Y from the reduction of the prediction error of Y when including the past of X was formulated by Wiener [1] and formalized by Granger [2]. While Granger provided a measure to study causal interactions for Gaussian linear processes, the underlying concept (Granger causality) can be used to derive a general criterion to test for causality [3]. In particular, in the framework of information theory, transfer entropy [4] extends the measure proposed by Granger so that it is applicable to stationary and non-stationary finite-order Markov processes [5].

We here show that the concept of Granger causality can be used to reliably determine between which nodes in a network causal interactions exist, but in contrast to commonly held beliefs, it is not adequate to quantify the strength of these causal interactions. Instead we propose a two-step procedure to infer and quantify causality. First, a Granger causality based measure, like transfer entropy, is applied to build a directed graph indicating causal interactions between the nodes. Second, for nodes fulfilling some conditions we describe, an alternative measure is used to quantify the strength of the causal interactions.

This procedure is exemplified with bivariate stochastic processes for which the information theoretic measures can be calculated analytically. This avoids dealing with the problem of the identification of the underlying processes from the recorded time series, and the problem of estimating the probability distributions which further difficult the analysis of causal interactions from experimental data. These simple examples allow us to illustrate some drawbacks of using the measures based on the concept of Granger causality in the way they are commonly applied for the study of neural causal interactions [e. g. 6-8].
